# Appraising MicroRNA-155 as a Noninvasive Diagnostic Biomarker for Cancer Detection: A Meta-Analysis

**DOI:** 10.1097/MD.0000000000002450

**Published:** 2016-01-15

**Authors:** Yi Hou, Jing Wang, Xianwen Wang, Shaomin Shi, Wanli Wang, Zhiying Chen

**Affiliations:** From the Department of Urinary Surgery (YH); Department of Respiratory Medicine, China-Japan Union Hospital of Jilin University, Changchun, China (JW, XW, SS, WW, ZC).

## Abstract

Cancer has been a major public health issue all over the world and cancer patients diagnosed at early stages have a comparatively favorable prognosis. The association between specific dysregulated expressed microRNA-155 (miRNA-155, miR-155) and tumorigenesis has been identified by numerous studies. However, perplexity and inconsistence arise from a wide range of studies due to heterogeneity. Therefore, this meta-analysis was carried out to validate the association between miR-155 and tumorigenesis together with the clinical applicability of miR-155.

Relevant studies were searched, identified, and selected from PubMed, Embase, Cochrane, Sinomed, and Wanfang database until July 5, 2015. Then, the sensitivity, specificity, and area under the summary receiver operator characteristic curve (AUC) were calculated to assess the overall performance miR-155 for cancer detection.

A total of 25 studies were included in the meta-analysis with a total number of 1896 cancer patients and 1226 healthy controls. The overall sensitivity and specificity was 76.8% (95%CI: 71.1–81.7%) and 82.9% (95% CI: 77.5–87.3%), respectively. In addition, the pooled AUC and partial AUC was 0.867 and 0.718, respectively. Results from subgroup analyses suggested that the diagnostic accuracy of miR-155 in the Caucasian group was significantly higher than that in the Asian group. Similarly, serum sample type may provide better diagnostic value of miR-155 than plasma. Apart from that, miR-155 in breast cancer achieved the highest accuracy compared with miR-155 in other types of cancer.

Results from meta-analysis suggested that miR-155 had great potential as a novel noninvasive biomarker for human cancer detection, particularly when breast cancer or Caucasian is involved. However, well-designed cohort or case control studies with large sample size should be implemented to confirm the diagnostic value of miR-155.

## INTRODUCTION

As the major public health problem in the worldwide, cancer is currently the second leading cause of death in the United States and is anticipated to exceed heart diseases as the leading cause of death in the future.^1^ Furthermore, ∼1.6 million new cancer cases and more than half million cancer deaths are predicted to occur in the United States in 2015.^2^ Non-small-cell lung cancer, which accounts for 80% of all lung cancer, is usually diagnosed at advanced stages leading to an overall 5-year survival rate of 0% to 40%. However, the 5-year survival rate will increase to 83% as long as the patients diagnosed at stage I.^3^ Similarly, the early detection of prostate cancer, oesophageal squamous cell carcinomas, breast cancer, and so on can remarkably increase the survival rate and reduce the morality.^4^ Hence, it is urgent to find a new method for the early diagnosis of various malignant tumors.

Recently, molecular biomarkers have gained great attention and numerous studies have revealed the critical role of microRNAs (miRNAs) in the development of cancers and proposed miRNAs as potential biomarkers for cancers diagnosis and therapy.^4–6^ MiRNAs are noncoding RNAs, and evolutionarily conserved that pleiotropically regulate gene expression at the post-transcriptional level.^7^ It has been proved that miRNAs independently or cooperatively interfere with various physiological and pathological processes, including hematopoietic lineage differentiation, proliferation, apoptosis, and oncogenesis.^8–10^ Besides that, tumor-specific miRNAs have enormous advantages over conventional cancer detection methods, including high stability, easy accessibility, and noninvasiveness. As a result of this, the promising role of miRNAs in the field of cancer detection has been hypothesized for a lot years.

In particular, microRNA-155 (miRNA-155, miR-155) regarded as one of the most familiar onco-miRNAs, and aberrant expression of miR-155 was reported in breast cancer, nonsmall cell lung cancer, and B-cell lymphoma.^11–13^ However, due to ethnicity, sample types and cancer types, there was not a comprehensive conclusion for the diagnostic value of miR-155 in detecting cancers. To summarize the results of a number of randomized controlled trials, we conducted a systematic meta-analysis to assess the diagnostic value of miR-155 for cancer detection.

## MATERIALS AND METHODS

### Search Strategy and Study Selection

A thorough search of relevant articles from PubMed, Embase, Cochrane, Sinomed and Wanfang database until July5, 2015, was performed using the following medical subject headings terms: (“neoplasms” or “cancer” or “tumor” or “malignancy”) and (“microRNA-155” or “miRNA-155” or “miR-155”) and (“diagnoses” or “ROC curve” or “sensitivity” or “specificity”). Also, manual retrieval was conducted to reduce selection bias. The ethics committee was not appropriated in this study.

Eligible studies should be strictly in accordance with the following criteria: (1) studies assessing the miR-155 expression profiling for cancer diagnosis; (2) cancer patients should be confirmed by a golden standard test; (3) sufficient data is available to derive the diagnostic two-by-two tables (true/false positive, true/false negative). The following exclusion criteria were considered: (1) studies investigating survival or prognosis of cancer; (2) conference report, editorials, letters, or reviews; (3) studies containing duplicate data and unqualified data. Studies were reviewed, screened, and selected by 2 independent reviewers using the above inclusion and exclusion criteria.

### Data Extraction and Quality Assessment

The full text and the additional information of each study were carefully reviewed. After that, the following data were extracted from each study: research details (first author, published year, and country of participant), study population characteristics (ethnicity, number of subjects, gender ratio, mean age, cancer types, and source of control), and relevant data for meta-analysis (specimen, sensitivity, specificity, data of two-by-two tables).

A quality assessment of individual studies was conducted using the criteria set by QUADAS-2.^14^ Each item on the QUADAS-2 list will be checked, and answered with yes, no or unclear. Finally, the scores of QUADAS-2 were recorded to determine the overall quality of selected studies.

### Statistical Methods

The random-effects model was used to evaluate the sensitivity and specificity together with their 95% confidence intervals (CIs). Moreover, the summary receiver operator characteristic (SROC) curve was generated and the corresponding area under the SROC curve (AUC) together with partial AUC was calculated to evaluate the accuracy of miR-155 in cancer detection. An AUC of 1.0 implies that the test has perfect diagnostic accuracy whereas an AUC of 0.5 indicates poor diagnostic accuracy.^15^ The difference in the diagnostic accuracy may result from the random error or significant heterogeneity among individual studies. Therefore, a chi-square test was performed to assess whether or not significant heterogeneity exists. If the *P* value of the chi-square test is <0.05, then there is significant heterogeneity among individual studies. As a result, a random-effects model will be adopted. Otherwise, the fixed-effects model will be applied in the meta-analysis. Subgroup analyses were also conducted to explore the potential sources of between-study heterogeneity. Furthermore, publication bias was estimated by Deek's funnel plot and a *P*-value of <0.05 indicates significant publication bias.^16^ All statistical analyses were performed using the R 3.1.2 software.

## RESULTS

### Included Studies

**FIGURE 1:**
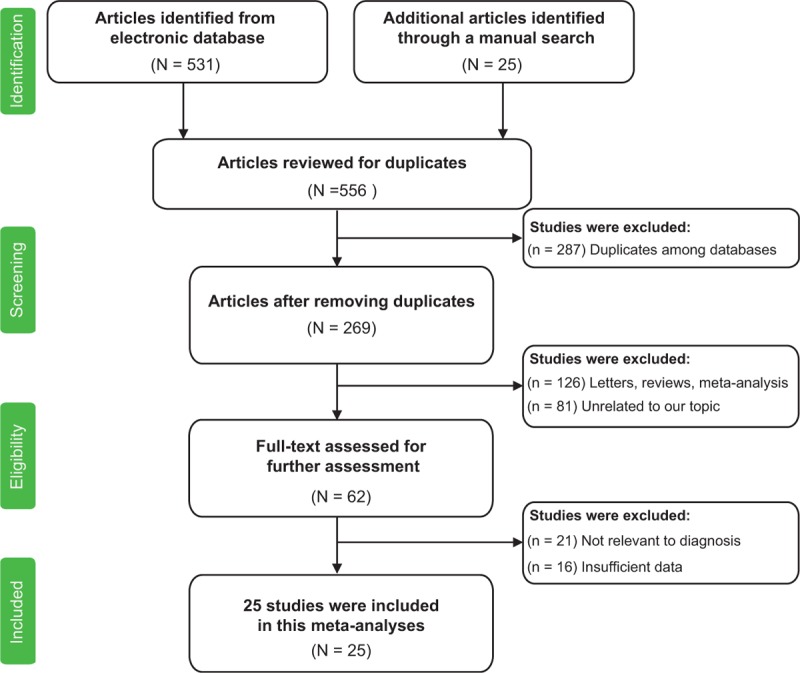
The flowchart of literature selection.

Figure 1 illustrates the process of article retrieval and study selection. Initially, 556 manuscripts were identified from various databases and 287 of them were excluded due to duplications. After titles and abstracts were reviewed, 207 of the remaining 269 articles were excluded: 126 articles were reviews, letters, and meta-analyses and 81 articles were not related to the research topic. As a result, 62 articles were suitable for full-text review. However, 37 of the 62 articles were further excluded: 21 manuscripts were not related to cancer diagnose and 16 manuscripts did not contain sufficient data. Finally, 25 articles were included in the final meta-analysis.^17–41^

### Study Characteristics and QUADAS Score

The main clinical features of the included studies were extracted and listed in Table 1 by order of publication year. A total of 3122 subjects from 25 studies (Asian: n = 15, Caucasian: n = 10) between 2009 and 2015 (1896 cancer patients and 1226 healthy controls) were included in our meta-analysis and each study had different specimen for cancer diagnose. All cancer cases in the study were confirmed by a pathological examination. In addition, different types of cancer were recorded including pancreatic cancer (n = 2), pancreatic ductal adenocarcinoma (n = 2), nonsmall cell lung cancer (n = 2), lung cancer (n = 4), diffuse large B cell lymphoma (n = 1), oesophageal squamous cell carcinoma (n = 1), breast cancer (n = 6), acute myeloid leukemia (n = 2), colorectal cancer (n = 1), papillary thyroid cancer (n = 1), oral squamous cell carcinomas (n = 2), and nasopharyngeal carcinoma (n = 1). The specimen types contained serum (n = 11), plasma (n = 8), tissues (n = 3), urine (n = 1), feces (n = 1), and sputum (n = 1).

**TABLE 1:**
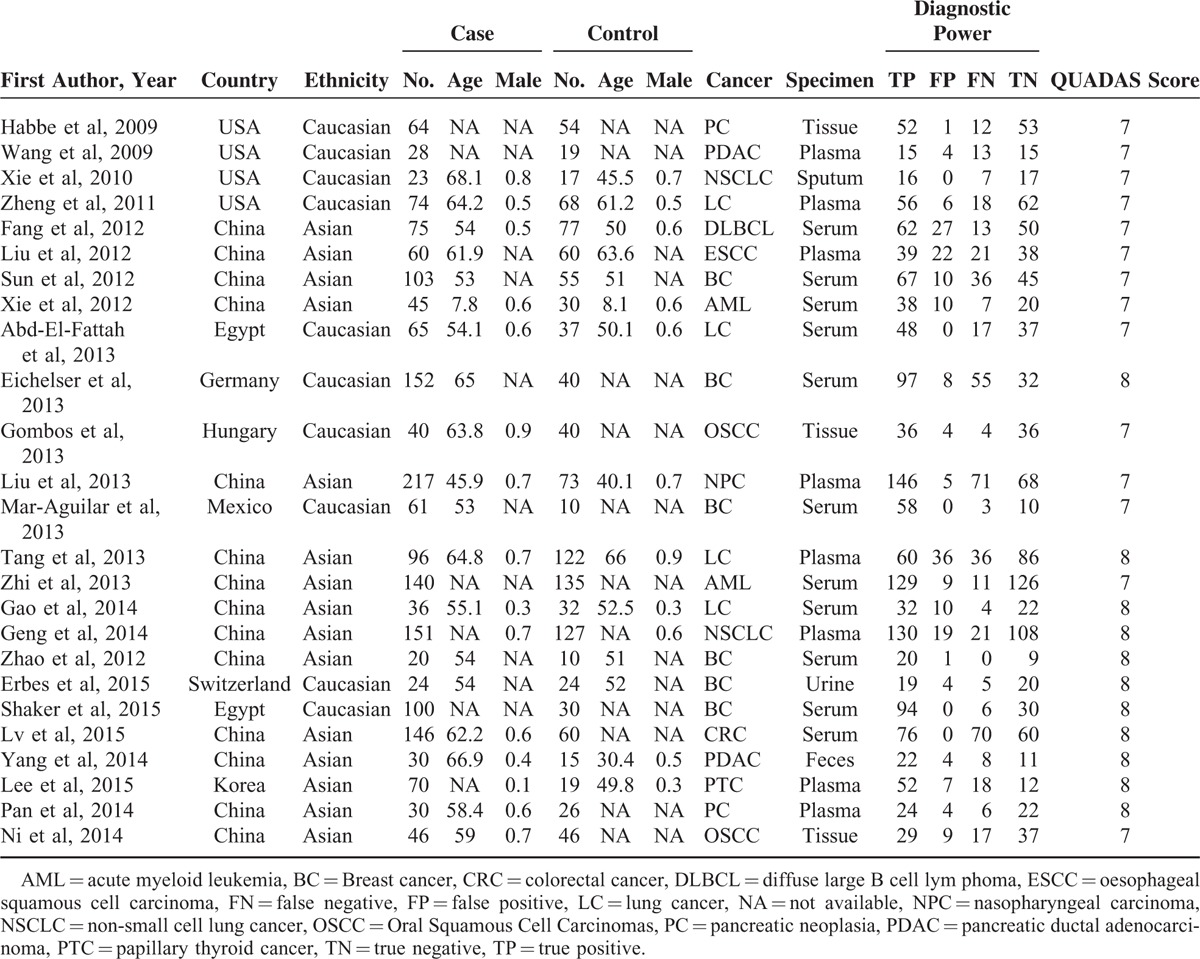
Main Characteristic of the Included Literatures in this Meta-Analysis

**FIGURE 2:**
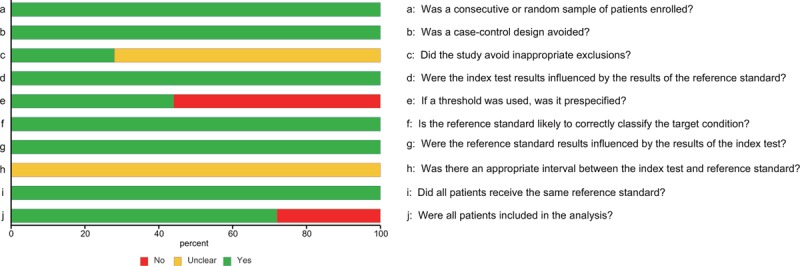
The result of QUADAS score for quality assessment.

Quality of the 25 studies was evaluated by QUADAS-2 and the majority of studies scored more than 7 out of 10. As a result, significant bias was not presented in the meta-analyses as suggested by Table 1 and Figure 2, and the detailed information of QUADAS-2 assessment was represented in Table S1.

### Diagnostic Accuracy and Subgroup Analyses

**FIGURE 3:**
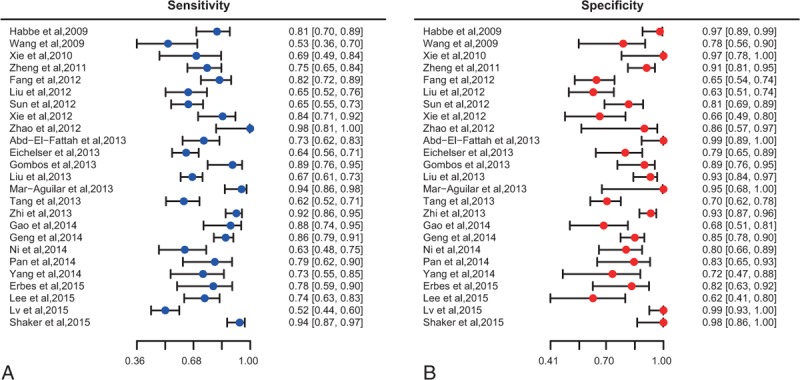
Forest plots of sensitivity and specificity.

**FIGURE 4:**
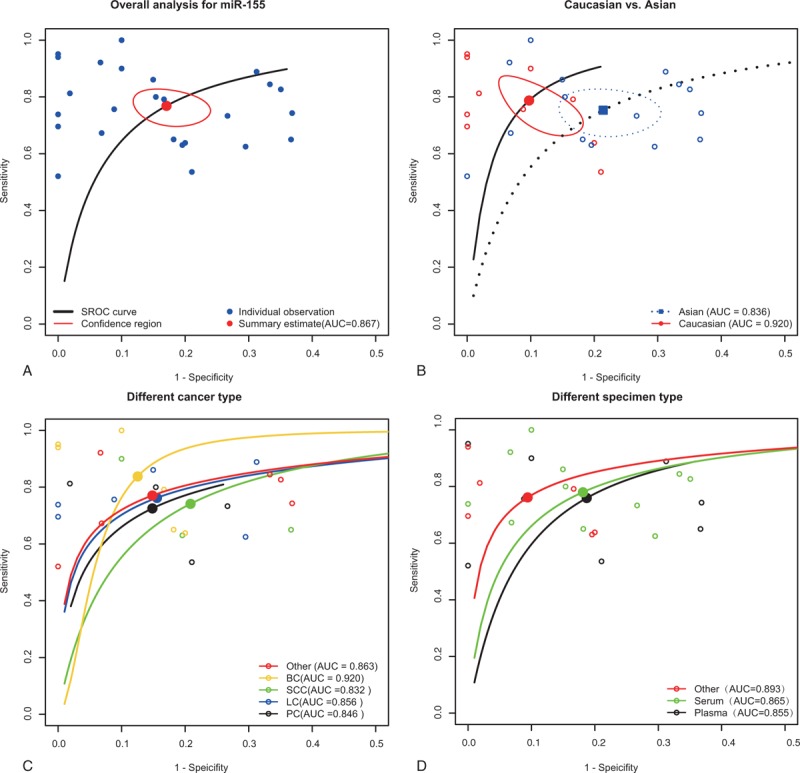
Summary ROC curve for miR-155 and subgroup analysis.ROC = receiver operator characteristic.

Figure 3 indicates the forest plots of sensitivity and specificity for individual studies and Table 2 revealed the corresponding sensitivity, specificity, AUC, and partial AUC. There was significant heterogeneity of sensitivity and specificity between individual studies as suggested by the chi-square test (both *P* < 0.05). Hence, the random effects model was used in the meta-analysis to evaluate the pool estimates. The overall pooled results for sensitivity and specificity were 76.8% (95%CI: 71.1–81.7 %) and 82.9% (95% CI: 77.5–87.3%), respectively. The SROC curve of miR-155 was indicated in Figure 4A with an overall AUC of 0.867 and partial AUC of 0.718.

**TABLE 2:**
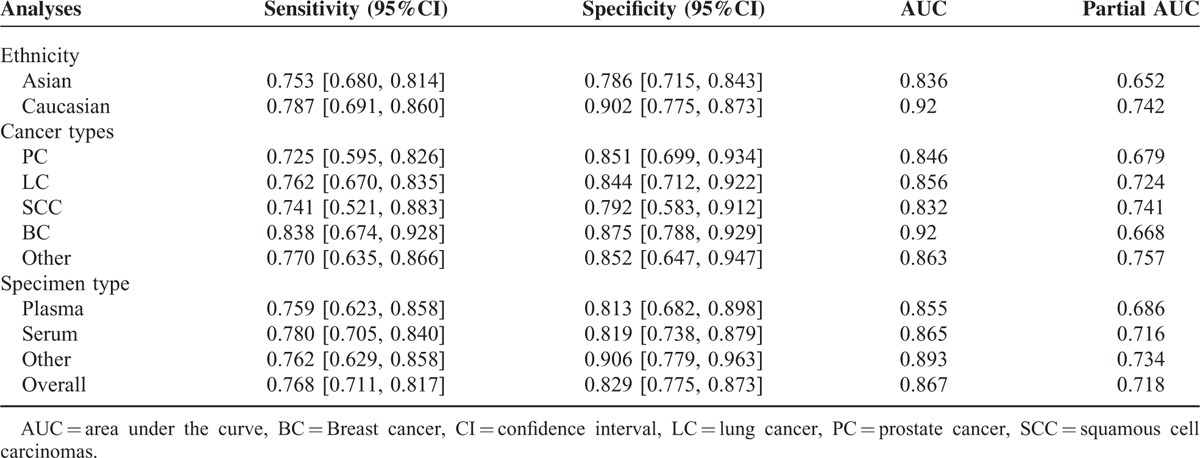
Overall and Subgroup Analyses of the Included Studies

Furthermore, subgroup analyses were conducted based on ethnicity, cancer types, and specimen types (Table 2). For studies based on Caucasian, the pooled sensitivity, specificity, AUC, and partial AUC was 0.787, 0.902, 0.92, and 0.742, respectively (Figure 4B). For studies based on Asian, the corresponding pooled estimates were 0.753, 0.786, 0.836, and 0.652 respectively (Figure 4B). Our results suggested that cancer detection using miR-155 was more accurate in Caucasian than in Asian. Besides, we also conducted a subgroup analysis according to different cancer types: prostate cancer, lung cancer, squamous cell carcinomas, breast cancer, and other types of cancer (nonsmall cell lung cancer, diffuse large B cell lymphoma, nasopharyngeal carcinoma, acute myeloid leukemia, papillary thyroid cancer, colorectal cancer) (Figure 4C). The pooled sensitivity and specificity together with their 95% CI of 5 cancer types were illustrated in Table 2 (prostate cancer: 0.725, 0.851; lung cancer: 0.762, 0.844; squamous cell carcinomas: 0.741, 0.792; breast cancer: 0.838, 0.875; and other types cancer: 0.770, 0.852) In addition, AUC and partial AUC of 5 cancer types were listed in Table 2 (prostate cancer: 0.846, 0.679; lung cancer: 0.856, 0.724; squamous cell carcinomas: 0.832, 0.741; breast cancer: 0.92, 0.668; other types of cancer: 0.863, 0.757). The highest sensitivity, specificity, AUC, and partial AUC in breast cancer suggested that miR-155 was more accurate in breast cancer diagnosis. Meanwhile, the subgroup analyses based on specimen types indicated that serum had relatively accurate diagnostic value compared to plasma with a sensitivity of 0.780 versus 0.759, AUC of 0.865 versus 0.855, and partial AUC of 0.716 versus 0.686 (Figure 4D).

### Publication Bias

**FIGURE 5:**
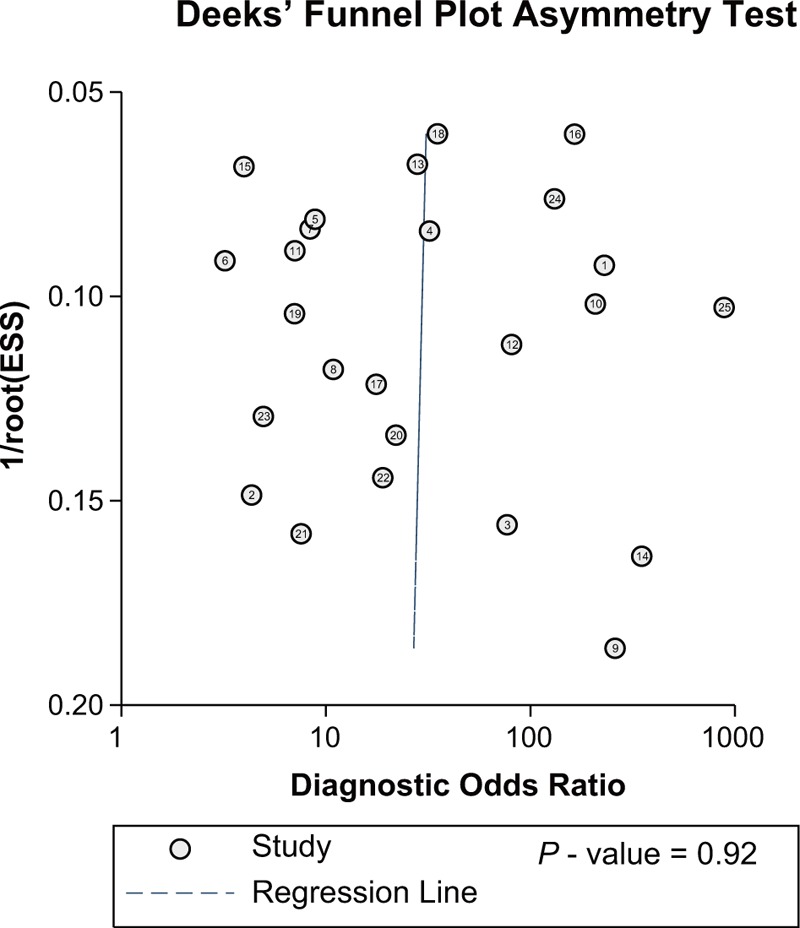
The publication bias of all included studies.

As suggested by Figure 5, there was no significant publications for included studies (*P* = 0.921) and therefore we did not have sufficient evidence to conclude a biased effect size.

## DISCUSSION

Cancer has become a major threat to both developed and developing countries due to its considerably high mortality and the increasing number of death over the last decades.^42^ According to the cancer statistics, the number of new cancer cases in the last 3 years increased by 6% per year over the world.^42–44^ Conventional cancer detection methods focus on the histological evaluation which rarely enables us to detect various cancers in their early stages. Other methods such as computed tomography, endoscopic ultrasound-guided fine needle aspiration and cytological analysis of sputum had a lot of limitations including late diagnosis, low diagnostic accuracy, and invasion to human body. It is surmised that cancer is attributed to the exogenous or intrinsic genetic alterations of cells and environmentally-induced genomic changes, which are not necessarily followed by protein dysfunctions or immediate structural changes.^45,46^ Therefore, it is urgent to make a breakthrough in the field of cancer detection in order to ensure early diagnose of cancer and timely implementation of intensive treatment.

As it was difficult to diagnose cancer in their early stages through histological evaluation, molecular biomarkers may serve as ideal cancer detection tools. Numerous studies have supported the promising role of miRNAs obtained from body fluid in cancer detection.^47–49^ According to these miRNAs detection research, a qualified miRNA need to fulfill the following suggested requirements: (1) their dysregulation expression in cancer was caused by molecular structural alteration; (2) phenotypic alteration were influenced by manipulation of the miRNA in vitro; and (3) target at least 1 cellular cancer gene. Based on these requirements, miR-155 was selected as numerous researches have indicated the altered expression of miR-155 in various cancer.^5,50,51^

Our meta-analysis suggested that the overall diagnostic accuracy of miR-155 was reliable with a pooled sensitivity of 0.768, a pooled specificity of 0.829, AUC of 0.867, and partial AUC of 0.718. Published researches had confirmed the use of miR-155 expression as biomarker for cancer detection, in which the diagnostic accuracy of miR-155 was highlighted. Recently, Wang et al reported that miR-155 has the potential diagnostic value for breast cancer detection, with a pooled sensitivity of 0.79 and specificity of 0.85.^52^ Wang et al also provided evidence that miR-155 could predict the prognosis and lymphatic invasion of nonsmall cell lung cancer.^53^ Apart from that, Wu et al revealed that miR-155 is likely to be used in a variety of cancer screening tests, with a pooled sensitivity of 0.76 and specificity of 0.82.^54^

Furthermore, subgroup analyses by ethnicity, cancer types, and specimen types were performed in the meta-analysis. Results of subgroup analyses suggested a significantly better diagnostic accuracy in Caucasian than that in Asian, with a pooled sensitivity of 0.787, specificity of 0.902, and AUC of 0.92. Similar results from Wu et al also revealed that miR-155 had more promising accuracy for cancer diagnosis in Caucasian than that in Asian. Moreover, Glas et al suggested that miRNA expression profiling might be more precise in the Caucasian population than that in the Asian population.^55^ The above evidence confirmed that using miR-155 as a biomarker for cancer detection in Caucasian could be more accurate than that in Asian.

In addition, the diagnostic accuracy in different cancer types had a lot of variation and the most accurate diagnose was found in detecting breast cancer with a sensitivity of 0.838, specificity of 0.875, and an AUC of 0.92. Wang et al suggested that the circuiting miR-155 has a potentially high diagnostic value with a sensitivity of 0.787 and specificity of 0.902 for breast cancer detection according to the current evidence, which is having better diagnostic value than mammography.^52^ Zhang et al revealed that mammography alone have a sensitivity of 0.578 and a specificity of 0.631.^56^ According the study of Roberts et al, prostate specific antigen (PSA) have a high sensitivity of 90% and a specificity of 30%; however, miR-155 had a more balanced diagnostic value with a sensitivity of 0.725 and a specificity of 0.851 than PSA.^57^ Toyoda revealed a higher sensitivity and specificity (0.886 and 0.926) for low-dose CT, which indicated that low-dose CT is more accurate than miR-155, whereas the diagnostic objects are a high-risk group for lung cancer, which will overestimate the diagnostic value of low-dose CT.^58^

Similarly, miR-155 in serum had more precise diagnostic value than that in plasma and this may be explained by the coagulation process which could affect the extracellular miRNA spectrum in the blood, resulting in different miRNA expression levels for various specimens.^59^ However, the analysis based on other specimen types contained only 6 studies and the small sample size could yield biased results, which could further impact the clinical conclusion. Besides that, Caucasian was involved in most of our included studies whereas Asian was involved in a small number of the included studies. Therefore, further large-size studies among Asian should be designed to provide a comprehensive result. Furthermore, some researchers insisted that tumorigenesis was very complex in which a panel of certain miRNAs was involved. Several studies included in our meta-analysis also suggested that combining miR-155 with certain miRNAs might yield a desirable diagnostic accuracy. However, choosing the optimal combination of miRNAs and the inconvenience for clinical routine detection constrain the development of miRNAs combination assays. Apart from that, how miRNAs mechanism affect tumorigenesis is an important bewilderment that must be solved. A study by Sun et al supported that miR-155 decreased endogenous FOXO3a protein, which is a well-studied tumor suppressor transcriptional factor and resides in the nucleus to transcribe pro-apoptotic genes.^23^ Another study by Kong et al suggested that miR-155 acts in transforming growth factor β-induced epithelial-mesenchymal transition by targeting the Rho family small GTPase RhoA transcript.^60^ Also, there is research showing that miR-155 influences the apoptosis by directly downregulates one of the MYC antagonists.^23^ As suggested by numerous studies, miR-155 may affect cell cycle by regulating some known oncogenes or tumor suppressor genes and even itself can act as oncogenes or tumor suppressor genes in carcinogenesis.^61,62^ Further fundamental studies should be designed to explore the mechanism of miR-155.

Although our meta-analysis yielded an encouraging result of miR-155 for cancer detection, several issues should be taken into account before suggesting any clinical conclusion. First, different normalization strategies and the lack of assurance for accurate measurement may impede the development and progression of miRNAs as biomarkers. Second, different concentrations of miR-155 were observed in different race, specimen types, and treatment status. These discrepancies caused by various genetic background, environmental factors, and resection surgery of tumor tissues could have substantial impact on the diagnostic accuracy of miR-155.

There also seemed to be some limitations in the meta-analysis. Results from the meta-analysis were based on unadjusted estimates because some studies did not provide detailed information to calculate the adjusted estimates. For example, we were not able to calculate the adjusted estimates due to the lack of miR-155 expression levels in different tumor stages. Besides that, a lot of confounding factors such as ethnicity, sample size, specimen types, and cancer types were not controlled which could result in biased statistical results. Despite of these limitations, our meta-analysis was probably the first one which concluded that miR-155 displays excellent characteristics in cancer detection.

In conclusion, our meta-analysis suggested that miR-155 has strong potential to be considered as a novel noninvasive biomarker for detection of human cancer. It is encouraged that studies with large sample size and matched case-controls should be designed in order to verify the diagnostic value of miR-155 in cancer detention.
